# Huh-7 cell line as an alternative cultural model for the production of human like erythropoietin (EPO)

**DOI:** 10.1186/1479-5876-9-186

**Published:** 2011-11-01

**Authors:** Humera Kausar, Sana Gull, Bushra Ijaz, Waqar Ahmad, Muhammad Tahir Sarwar, Zafar Iqbal, Zafar Nawaz, Sheikh Riazuddin, Sajida Hassan

**Affiliations:** 1 Centre of Excellence in Molecular Biology, University of the Punjab, Lahore Pakistan; 2 Department of Human Genetics, Nijmegen Centre for Molecular Life Sciences, Radboud, University Nijmegen Medical Center, Nijmegen, Netherlands; 3 Department Biochemistry & Molecular Biology, University of Miami, USA

**Keywords:** EPO, erythropoietin; CHO, Chinese hamster ovary cell line; Huh-7, Human hepatoma cell line; PCR, polymerase chain reaction

## Abstract

**Background and Aims:**

Erythropoietin (EPO) is a glycoprotein hormone which is required to regulate the production of red blood cells. Deficiency of EPO is known to cause anemia in chronically infected renal patients and they require regular blood transfusion. Availability of recombinant EPO has eliminated the need for blood transfusion and now it is extensively used for the treatment of anemia. Glycosylation of erythropoietin is essential for its secretion, stability, protein conformation and biological activity. However, maintenance of human like glycosylation pattern during manufacturing of EPO is a major challenge in biotechnology. Currently, Chinese hamster ovary (CHO) cell line is used for the commercial production of erythropoietin but this cell line does not maintain glycosylation resembling human system. With the trend to eliminate non-human constituent from biopharmaceutical products, as a preliminary approach, we have investigated the potential of human hepatoma cell line (Huh-7) to produce recombinant EPO.

**Materials and methods:**

Initially, the secretory signal and Kozak sequences was added before the EPO mature protein sequence using overlap extension PCR technique. PCR-amplified cDNA fragments of EPO was inserted into mammalian expression vector under the control of the cytomegalovirus (CMV) promoter and transiently expressed in CHO and Huh-7 cell lines. After RT-PCR analysis, ELISA and Western blotting was performed to verify the immunochemical properties of secreted EPO.

**Results:**

Addition of secretory signal and Kozak sequence facilitated the extra-cellular secretion and enhanced the expression of EPO protein. Significant expression (*P *< 0.05) of EPO was observed in the medium from Huh-7 cell line.

**Conclusion:**

Huh-7 cell line has a great potential to produce glycosylated EPO, suggesting the use of this cell line to produce glycoproteins of the therapeutic importance resembling to the natural human system.

## Introduction

EPO is an essential growth factor required for the proliferation and differentiation of the stem cells that produce red blood cells [1]. Structurally, EPO is a glycoprotein contains approximately 40% carbohydrate [2]. These carbohydrate structures are very essential for many biological properties like pharmacokinetics, secretion, stability, receptor recognition and antigenicity, protein conformation and biological activity [3].

Kidney is the main production unit of EPO in normal person [4]. Any damage to kidney tissues abolishes the EPO secretion from kidney thus causing anemia in renal patients. Recombinant DNA technology has enabled manufacture of the recombinant human EPO (rHuEPO) to use as a drug. The initial findings with the use of rHuEPO for the treatment of anemia were so inspiring that it was licensed to use as therapy within three years of its availability [5]. Now the drug is amongst the top selling pharmaceutical product worldwide and considered applicable for a variety of disorders such as anemia associated with renal failure, hepatitis C infection, cancer, human immunodeficiency virus infections, and cardiovascular disease [6].

The glycosylation pattern of protein is affected by several parameters including the protein structure [7], host system used to produce glycoprotein [8] and the culture conditions [9]. Among all these parameters, host system greatly affects the glycosylation pattern, thus selection of appropriate host system is very crucial to produce the therapeutic glycoprotein. Among the prokaryotic and eukaryotic expression systems only mammalian cell lines have the capacity to carry out proper glycosylation. Currently, the mammalian system used to produce the rHuEPO is CHO cell line [10] associated with some disadvantages e.g. it is not able to control the human like glycosylation. EPO produced from CHO cells contains *N*-glycolylneuraminic acid [11], but humans lack the pathway for the synthesis of *N*-glycolylneuraminic acid [12], thus recombinant EPO that contain *N*-glycolylneuraminic acid are subjected to clearance by anti-*N*-glycolylneuraminic acid antibodies present in human serum [13]. Therapeutic use of such glycoproteins may cause undesirable responses that can affect the efficacy of the treatment. So the use of human cell line for therapeutic glycoproteins production is unavoidable. Human-cell based expression system can provide some unique characteristics to biopharmaceutics including lower immunogenicity, greater biological activity and increased half life.

With the trend to introduce the human-cell-based expression system to synthesize the biopharmaceutical products, several human cell lines have been proposed *e.g*. Human embryonic kidney (HEK293) and human fibro-sarcoma (HT-1080) cell line [14]. In the present study, we have used Huh-7 cell line for the first time for expression study of EPO gene in comparison to CHO cell line. Previously this cell line was used for the expression study of insulin [15,16]. Huh-7 cell line is derived from epithelial cells of human liver. We hypothesized that EPO produced from human liver cell line would be properly folded and glycosylated, as EPO is mainly produced by liver cells during the fetal stage [17].

## Materials and methods

### Construction of vectors for erythropoietin gene expression

**Figure 1:**
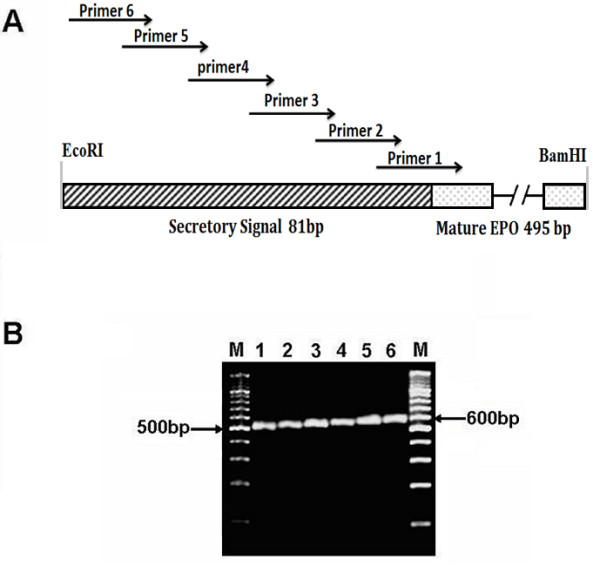
**Construction and PCR confirmation of the expression vector of EPO after the addition of secretory signal by using overlapping primers**. **(A) **Schematic diagram showing the signal sequence addition using six overlapping primers before the 495 bp of mature EPO. Sequences of primers are given in Table 1. **(B) **Agarose gel electrophoresis showing step wise addition of secretory signal. M, 100bp DNA ladder; Lane 1-6 sequential addition of secretory signal sequence, after every PCR 15-18 bp of the secretory signal is added. The final 588 bp (including restriction sites) fragment of the EPO fused with 81 bp of signal peptide is achieved.

**Figure 2:**
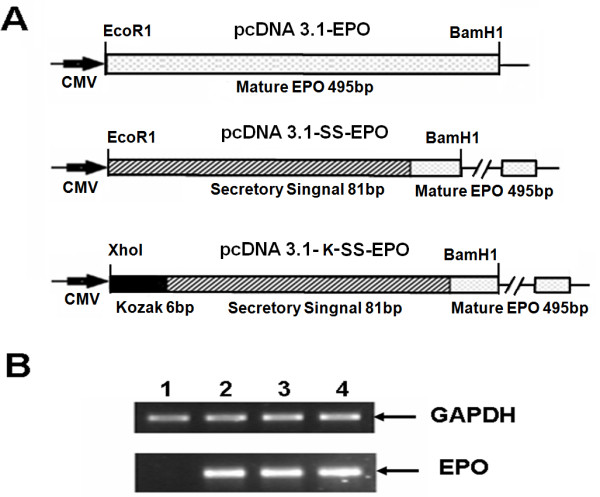
**EPO cDNA constructs and their expression analysis in CHO cell line**. **(A) **Schematic diagram showing the three EPO expression vectors pcDNA3.1-EPO (without secretory signal, having only mature EPO protein sequence of 495 bp), pcDNA3.1-SS-EPO (with secretory signal fused with mature EPO protein sequence) and pcDNA3.1-K-SS-EPO (having additional six nucleotide of Kozak sequence (GCCACC) before ATG start codon). CMV promoter region of pcDNA 3.1 vector is also shown. **(B) **mRNA expression analysis showing equal level of expression of all three vectors. 1 μg of the total RNA extracted from CHO cells after 24 hours post transfection was subjected to reverse transcriptase PCR. Lane 1, CHO cells as mock Lane 2, 3 and 4 showing mRNA expression of pcDNA3.1-EPO, pcDNA3.1-SS-EPO and pcDNA3.1-K-SS-EPO respectively. GAPDH is used as internal control. All experiments were performed in duplicate.

EPO-cDNA construct pcDNA3.1-EPO containing only mature EPO protein sequence (495 bp) was provided by CEMB (Centre of Excellence in Molecular Biology, University of the Punjab, Lahore). For the construction of EPO secretory plasmid, we used this plasmid as a starting material. PCR-based addition of secretory signal was performed for extra-cellular protein secretion using the six over lapping primers (Table 1) at 5' end of EPO-cDNA construct. Final PCR product was a fragment of 588 bp, having 495 bp of mature EPO protein sequence, 81 bp of secretory signal peptide and 12 bp of *EcoR1 *and *BamH1 *restriction sites at 5' and 3' end respectively (Figure 1). PCR-product was digested with *EcoR1 *and *BamH1 *(Fermentas, USA) and used for ligation into plasmid pcDNA3.1/zeo the resulting plasmid was named as pcDNA3.1-SS-EPO (Figure 2A).

**Table 1:** Primer sequences

Signal sequence and Kozak addition	
Primer 1	CTCCCAGTCCTGGGCGCCCCACCACGCCTC
Primer 2	TCGCTCCCTCTGGGCCTCCCAGTCCTGGGC
Primer 3	CTCCTGTCCCTGCTGTCGCTCCCTCTGGGC
Primer 4	GCCTGGCTGTGGCTTCTCCTGTCCCTGCTG
Primer 5	GTGCACGAATGTCCTGCCTGGCTGTGGCTT
Primer 6	GAATTC**ATG**GGGGTGCACGAATGTCCTGCC
Primer K	CTCGAGGCCACC**ATG**GGGGTGCACGAATGT
Primer R	GGATCCTCAGTCCCCTGTCCTGCAGGC

RT-PCR	

Forward	AGTTAATTTCTATGCCTGGAAGAGG
Reverse	GGAAAGTGTCAGCAGTGATTGTT
GAPDH-F	ACCACAGTCCATGCCATCAC
GAPDH-R	TCCACCACCCTGTTGCTGTA

In another approach six nucleotides of Kozak sequence (GCCACC), was also added before the start codon of EPO-cDNA construct by using the primer K as forward and primer R as reverse primers (Table 1). The size of this fragment was 594 bp including the *Xho*1 and *BamH*1 restriction sites at 5' and 3' end respectively. PCR-product was digested with *Xho1 *and *BamH1 *and used for ligation into plasmid pcDNA3.1/zeo the resulting plasmid was named as pcDNA3.1-K-SS-EPO (Figure 2A). Following transformation and subsequent plasmid purification, of randomly selected clones, restriction digestion and sequencing analysis was performed using Big Dye chain termination reaction on an ABI 3100 DNA sequencer (Applied BioSystem) to verify the cloning of EPO-cDNA constructs in expression vector.

### Cell culture

Huh-7 cell line was maintained in 75 cm^2 ^culture flasks (Iwaki, Japan) containing DMEM (Sigma Aldrich, USA) supplemented with 100 μg/ml penicillin/streptomycin, and 10% FBS as complete culture media (CCM) (Sigma Aldrich, USA), at 37°C with 5% CO_2_. The CCM was renewed every third day and were passaged every 4-5 days. Viable cells were counted using 0.5% trypan blue (Sigma Aldrich, USA). Similarly CHO cell line was maintained in DMEM Ham's F12 (Sigma Aldrich, USA) and maintained as described for Huh-7 cells.

### Transient transfection

Approximately 3.5 × 10^5 ^CHO cells were seeded into 60 mm plates and cultured in CCM until 60-80% confluent prior to transfection. 8 μg of pcDNA3.1-EPO, pcDNA3.1-SS-EPO and pcDNA3.1-K-SS-EPO was transiently transfected in CHO cell line in serum-free media using Lipofectamine™ 2000 (Invitrogen) according to the manufacturer's protocol. After 6 hours incubation at 37°C in 5% CO_2_, cells were washed with 1x PBS and CCM was added to the cells. Cells were harvested at 24 hours post-transfection to analyze the EPO expression at mRNA and protein level. Similarly the Huh-7 cells were transfected as described above.

### RT-PCR (Reverse transcriptase PCR)

For EPO gene expression analysis, total RNA from Huh-7 and CHO cells transiently transfected with mock, EPO expression plasmids was extracted using Purescript RNA isolation kit (Gentra, USA) according to manufacturer's protocol. cDNA was synthesized using 1 μg of total RNA with SuperScript III enzyme (Invitrogen, USA). EPO gene expression analysis was carried out using RT-PCR on ABI 2700 PCR machine using gene specific primers. PCR conditions were as follow: 95°C, 30 sec; 57°C, 30 sec and extension at 72°C for 40 sec for 30 cycles. GAPDH was used as an internal control.

### ELISA (Enzyme Liked Immunosorbent assay)

For intra-cellular expression analysis of EPO, cells were harvested with ProteoJET mammalian cell lysis reagent (Fermentas, Canada) and for extra-cellular EPO expression supernatant was collected after 24 hours of transfection. Intra and extra-cellular EPO expression analysis was performed through ELISA (DRG, USA) according to manufacturer's protocol. Total 50 μg of intra-cellular protein and 200 μl of media for extra-cellular EPO expression analysis were subjected to ELISA. Absorbance was read at 450 nm on ELISA plate reader to determine the concentration of EPO in samples. The intensity of the yellow color developed was directly proportional to the concentration of EPO.

### Western Blotting

Mature erythropoietin is secreted into the medium, so supernatant was collected for comparative extra-cellular EPO expression analysis from CHO and Huh-7 cells, 24 hours post transfection. Samples and commercially available recombinant human erythropoietin (as a positive control) were subjected to electrophoresis on 12% SDS-PAGE and electrophoretically transferred to a nitrocellulose membrane using manufacturer's protocol (Bio-Rad, USA). Blots were incubated with specific-monoclonal antibodies of EPO (Santa Cruz Biotechnology, Inc, USA). Protein expression was evaluated using chemiluminescence detection kit (Sigma Aldrich, USA).

## Results and discussion

### Construction of secretory plasmid

**Figure 3:**
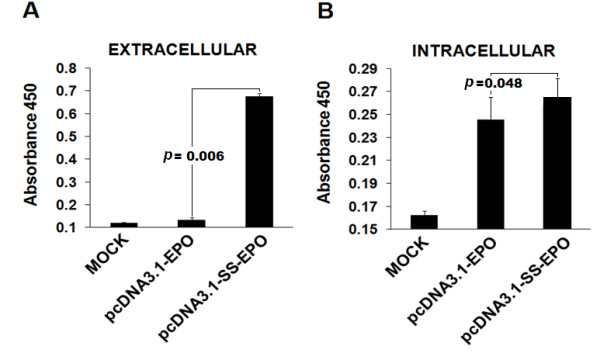
**Intra and extra-cellular expression of EPO after the addition of secretory signal sequence**. Total protein was extracted and subjected to ELISA. Optical density was measured at 450 nm. **(A) **Relative extra-cellular EPO expression showing that after the addition of signal peptide secretion of EPO into the medium is dramatically increased. **(B) **Comparison of intra-cellular EPO expression with (pcDNA3.1-SS-EPO) and without signal peptide (pcDNA3.1-EPO) expression vectors. Intra-cellular expression of protein is also slightly increased after the addition of signal peptide.

Recombinant proteins are targeted to the secretory pathway for post-translational modifications and proper folding. This is because of the fact that enzymes responsible for posttranslational modifications, e.g. glycosylation, are found within the endoplasmic reticulum and Golgi bodies [18]. Secretion of protein into the media especially of pharmaceutically importance facilitate the separation of specific protein from the intra-cellular pool of proteins, thus the purification and other downstream processing become easier. Keeping in view the importance of secretory signal we added the EPO's native secretory signal [19] at amino terminus of mature EPO protein. Using overlap extension PCR technique we successfully added 81 nucleotides of secretory signal at 5' end of mature EPO protein sequence (Figure 1). Finally, a fragment of 588 bp encoding signal peptide and EPO mature protein was cloned into pcDNA3.1/zeo (-ve) vector. The cloned fragment was sequenced to confirm the orientation and sequence of EPO gene by comparing with the reported EPO gene sequence. The secretion efficiency of the plasmid was evaluated by transfecting the both plasmids without secretory signal (pcDNA3.1-EPO) and with secretory signal (pcDNA3.1-SS-EPO) in CHO cell line. Equal numbers of cells (3.5 × 10^5^) were plated and same amount of both plasmid DNA (8 μg) were used for transfection. Twenty four hours post-transfection RNA was isolated and quantified using nanodrop (ND-1000). Total 1 μg of cellular RNA was reverse transcribed into cDNA and subjected to RT-PCR analysis. Results of RT-PCR showed that both plasmids (with and without signal sequence) transcribed the similar level of EPO mRNA. GAPDH gene was used as an internal control (Figure 2B). Intra- and extra-cellular EPO expression were analyzed through ELISA. We observed that EPO protein was hardly secreted in the medium when pcDNA3.1-EPO was transfected, although it was produced intra-cellularly (Figure 3). In comparison, medium samples obtained from cells transfected with pcDNA3.1-SS-EPO gave more absorbance that showed a significant amount of EPO secreted into the medium (*P *= 0.006). Interestingly, we also found that addition of secretory signal peptide not only facilitated the secretion of EPO but a slight increase in intra-cellular protein expression was also observed. These findings suggest that overall translation efficiency of EPO gene was also affected after the addition of secretory signal peptide. As Stenstrom *et al*. reported that the presence of initiation codon as well as context of initiation codon influence the level of gene expression [20]. Another possible explanation of the findings might be the proper recruitment of the EPO mRNA to the endoplasmic reticulum after the signal sequence addition. If the targeting is not fast enough, this may cause the decrease in protein synthesis and secretion [21].

### Effect of Kozak sequence on erythropoietin expression

**Figure 4:**
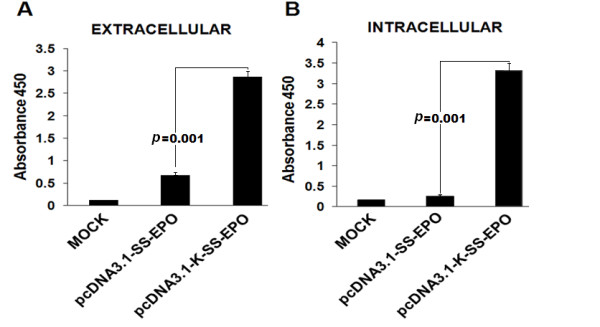
**Intra and extra-cellular expression analysis of EPO with and without Kozak sequence addition, through ELISA**. Optical density was measured at 450 nm. Both intra and extra-cellular EPO expression increased after the addition of Kozak sequence. **(A) **Comparison of extra-cellular EPO expression with Kozak (pcDNA3.1-K-SS-EPO) and without Kozak sequence (pcDNA3.1-SS-EPO) expression vectors. **(B) **Comparison of intra-cellular EPO expression after the addition of Kozak.

For the efficient production of recombinant proteins in mammalian expression system Kozak sequence could be used as a translation enhancer sequence [22]. After the confirmation of the functionality of secretory signal, we aimed to examine whether EPO expression is affected by adding Kozak sequence or not. For this purpose, we added the six nucleotides of Kozak sequence (GCCACC) before the start codon of EPO gene. We observed increased expression of EPO protein after the addition of Kozak sequence, as the higher amounts of intra-and extra-cellular EPO protein was obtained when pcDNA3.1-K-SS-EPO (*P *= 0.001) was transfected in CHO cell line compared to pcDNA3.1-SS-EPO (without Kozak) (Figure 4). In order to investigate whether the observed increased in expression level caused by the inclusion of Kozak sequence into EPO cDNA construct take place at the stage of transcription or translation, the levels of EPO mRNA in transfected cell populations were determined by semi quantitative RT-PCR. Our RT-PCR results showed that the both plasmids with and without Kozak transcribed the EPO mRNA with equal efficiency (Figure 2). As in both plasmids EPO gene construct was present under the control of same promoter *i.e*. CMV.

### Comparative expression analysis of EPO in CHO and Huh-7 cell line

**Figure 5:**
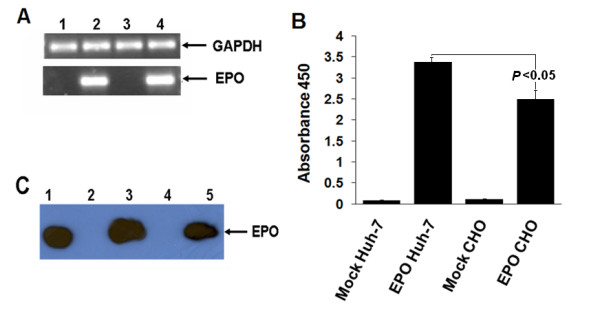
**Comparative expression analysis of EPO in CHO and Huh-7 cell line**. **(A) **mRNA expression analysis showing equal level of expression of pcDNA3.1-K-SS-EPO vector in CHO and Huh-7 cell line. 1 μg of the total RNA extracted from CHO and Huh-7 cells after 24 hours post transfection was subjected to reverse transcriptase PCR. Lane 1, mock Huh-7 cells; Lane 2, EPO gene expression in Huh-7; Lane 3, CHO cells as mock; Lane 4, EPO gene expressing in CHO cell line. GAPDH is used as an internal control. **(B) **Comparative expression analysis of extra-cellular level of EPO protein through ELISA in CHO and Huh-7 cell line. 8 μg of the pcDNA3.1-K-SS-EPO vector was transfected in Huh-7 and CHO cell line, 24 hours post transfection media was collected and 200 μl of media was loaded on EPO antigen coated ELISA plates. Graph is showing that Huh-7 cells secreted the increased amount of EPO and difference in expression level is significant (*P < 0.05*). **(C) **Extra-cellular protein expression levels were determined by Western blot analysis in CHO and Huh-7 cell lines using EPO specific antibodies. Lane1, Commercially available purified EPO protein as standard; Lane 2, Huh-7 cells as mock; Lane 3, EPO expressed from Huh-7cell line; Lane 4, CHO cells as mock; Lane 5, EPO expressed from CHO cells.

Prior to evaluate the potential of Huh-7 cell line to produce and secrete the EPO protein, we first compared the translation and secretion efficiency of all three EPO cDNA constructs in CHO cell line (Figure 3 and 4). We selected the plasmid pcDNA3.1-K-SS-EPO for comparative expression analysis of EPO gene in CHO and Huh-7 cell line as this plasmid displayed high levels of EPO translation and secretion efficiency because it contained the both translation enhancing elements *i.e*. secretory signal sequence and Kozak sequence. So to be able to compare EPO expression level in CHO and Huh-7 cell line, equal amount of plasmid was transiently transfected in both cell lines. EPO gene expression was analyzed at both mRNA and protein level. Our RT-PCR results showed approximately same amount of EPO mRNA transcribed from both cell lines (Figure 5A). ELISA analysis showed significant (*P *< 0.05) increase in EPO secretion in medium collected from Huh-7 cell line compared to CHO cell line (Figure 5B). One possible explanation for this increased expression of EPO from Huh-7 cell line is might be the effect of Kozak sequence. It is reported that effect of Kozak on protein expression varies among host systems [23]. Although, CHO cell line also showed increase expression level after the addition of Kozak sequence (Figure 4) but higher expression in Huh-7 cells suggest that Kozak consensus sequence might be more compatible with the human cell line. Further experiments, involving the other human and non-human but mammalian cell lines can be used to test this possibility. ELISA results were further confirmed by western blot analysis. Results of western blot verified the immunochemical properties of secreted EPO. EPO attains its size of 34 KDa after proper glycosylation during posttranslational modification whereas, unglycosylated form has lower molecular weight (18.4 KDa), indicated by smaller size protein on SDS-PAGE. In accord with this evidence, we observed a similar sized EPO protein (34 KDa) on Western blot when run side by side with commercially available glycosylated EPO as positive control suggesting that CHO and Huh-7 cell lines produced glycosylated form of EPO (Figure 5C) based upon their size similarity.

Our results showed that Huh-7 cells have the potential to secrete the comparable level of EPO to the CHO cell line which is already being used for commercial production of erythropoietin. Although, CHO cell line has several advantages including high growth rate and high productivity, but human cell-based expression is expected to produce EPO with post-translational modifications similar to their natural counterpart as some sugar tarnsfering enzymes are not present in CHO cell line e.g. α2-6 sialyltransferase and α1-3/4 fucosyltransferase [24].

In many studies people have used Huh-7 cell line as model system for expression studies of human proteins [25-28]. In the present study, Huh-7 cell line has shown the potential to produce the glycosylated EPO protein as an initial finding. However, to produce EPO at commercial level using Huh-7 cell line further characterization should be undertaken to determine the exact extent of sialylation, as it is reported that increased sialylation of EPO has been shown to increase its circulatory half-life. And this could be done through high pH anion exchange chromatography and isoelectric focusing.

Further studies to evaluate the bioactivity, up scalability and compatibility of Huh-7 cells with bioreactor are also needed. These findings will not only assist the biopharmaceutical industry by providing a human-cell-based expression system to produce recombinant glycosylated protein but, is also a preliminary step to make use of liver cells to deliver the EPO gene in chronically infected renal patients to cure anemia.

## Competing interests

The authors declare that they have no competing interests.

## Authors' contributions

HK and SG contributed equally to this work. BI, WA and ZI analyze the data and helped in paper write up. MTS maintained the cell cultures and carried out the cell culture experiments. ZN, SR and SH designed the study; they also checked the revised manuscript thoroughly and confirmed all the data given in manuscript. All work was performed under supervision of SH. We all authors read and approved the final manuscript.

## Authors' information

HK, BI and MTS are Ph.D. scholars in discipline of Molecular Biology at CEMB, University of the Punjab, Lahore, WA (M Phil Chemistry) and SG (MSc Biochemistry) are Research Officers; ZI is Ph.D. scholars at Radboud University Nijmegen Medical Center, Nijmegen, Netherlands; ZN (Biological Sciences) is Associate Professor in Biochemistry & Molecular Biology department, University of Miami, USA; while SH (PhD Molecular Biology) is Principal Investigator at CEMB, University of the Punjab, Lahore.
